# Perspectives of health care professionals on the facilitators and barriers to the implementation of a stroke rehabilitation guidelines cluster randomized controlled trial

**DOI:** 10.1186/s12913-017-2389-7

**Published:** 2017-06-26

**Authors:** Sarah E. P. Munce, Ian D. Graham, Nancy M. Salbach, Susan B. Jaglal, Carol L. Richards, Janice J. Eng, Johanne Desrosiers, Marilyn MacKay-Lyons, Sharon Wood-Dauphinee, Nicol Korner-Bitensky, Nancy E. Mayo, Robert W. Teasell, Merrick Zwarenstein, Jennifer Mokry, Sandra Black, Mark T. Bayley

**Affiliations:** 1 Toronto Rehabilitation Institute-University Health Network, 550 University Avenue, Toronto, Ontario M5G 2A2 Canada; 2 Centre for Practice-Changing Research, The Ottawa Hospital Research Institute, 501 Smyth Road, Box 711, Ottawa, Ontario K1H 8L6 Canada; 3 Department of Physical Therapy, University of Toronto, 160-500 University Ave, Toronto, Ontario M5G 1V7 Canada; 4 Department of Rehabilitation, Faculty of Medicine, Université Laval and Centre de Recherche en Réadaptation et Intégration Sociale (CIRRIS), Québec City, Quebec Canada; 5 Institut de Réadaptation en Déficience Physique de Québec (IRDPQ) Site Hamel, 525 Boul. Wilfrid-Hamel Est, Québec City, Quebec G1M 2S8 Canada; 6 University of British Columbia, 212 - 2177 Wesbrook Mall, Vancouver, BC V6T 1Z3 Canada; 7 Université de Sherbrooke, Faculty of Medicine and Health Sciences, 3001, 12e avenue nord, Bureau FM-2208, Sherbrooke, Québec J1H 5N4 Canada; 8 Office 405 Forrest Building, School of Physiotherapy, Dalhousie University, 5869 University Avenue, PO Box 15000, Halifax, Nova Scotia B3H 4R2 Canada; 9 McGill University, School of Physical and Occupational Therapy, 3630 Promenade Sir William Osler, Montreal, Quebec H3G 1Y5 Canada; 10 Division of Clinical Epidemiology, Division of Geriatrics, McGill University Health Center, Royal Victoria Hospital Site, Ross Pavilion R4.29, 687 Pine Ave West, Montreal, Quebec H3A 1A1 Canada; 11 Parkwood Institute, 550 Wellington Road, London, Ontario N6C 0A7 Canada; 12 Schulich School of Medicine & Dentistry, Western University, Western Centre for Public Health and Family Medicine, 1151 Richmond St, London, Ontario N6A 3K7 Canada; 13 Sunnybrook Health Sciences Centre, 2075 Bayview Avenue, Room A4 21, Toronto, Ontario M4N 3M5 Canada; 14 Neuro Rehabilitation Program, Toronto Rehabilitation Institute-University Health Network, 550 University Avenue, room 3-131 (3-East) 3rd Floor University Wing, Toronto, ON M5G 2A2 Canada

**Keywords:** Facilitators, Barriers, Implementation, Evidence-based recommendations, Clinical practice guidelines, Rehabilitation, Stroke, Qualitative

## Abstract

**Background:**

The Stroke Canada Optimization of Rehabilitation by Evidence Implementation Trial (SCORE-IT) was a cluster randomized controlled trial that evaluated two knowledge translation (KT) interventions for the promotion of the uptake of best practice recommendations for interventions targeting upper and lower extremity function, postural control, and mobility. Twenty rehabilitation centers across Canada were randomly assigned to either the facilitated or passive KT intervention. The objective of the current study was to understand the factors influencing the implementation of the recommended treatments and KT interventions from the perspective of nurses, occupational therapists and physical therapists, and clinical managers following completion of the trial.

**Methods:**

A qualitative descriptive approach involving focus groups was used. Thematic analysis was used to understand the factors influencing the implementation of the recommended treatments and KT interventions. The Clinical Practice Guidelines Framework for Improvement guided the analysis.

**Results:**

Thirty-three participants were interviewed from 11 of the 20 study sites (6 sites from the facilitated KT arm and 5 sites from the passive KT arm). The following factors influencing the implementation of the recommended treatments and KT interventions emerged: facilitation, agreement with the intervention – practical, familiarity with the recommended treatments, and environmental factors, including time and resources. Each of these themes includes the sub-themes of facilitator and/or barrier. Improved team communication and interdisciplinary collaboration emerged as an unintended outcome of the trial across both arms in addition to a facilitator to the implementation of the treatment recommendations. Facilitation was identified as a facilitator to implementation of the KT interventions in the passive KT intervention arm despite the lack of formally instituted facilitators in this arm of the trial.

**Conclusions:**

This is one of the first studies to examine the factors influencing the implementation of stroke recommendations and associated KT interventions within the context of a trial. Findings highlight the important role of self-selected facilitators to implementation efforts. Future research should seek to better understand the specific characteristics of facilitators that are associated with successful implementation and clinical outcomes, especially within the context of stroke rehabilitation.

**Electronic supplementary material:**

The online version of this article (doi:10.1186/s12913-017-2389-7) contains supplementary material, which is available to authorized users.

## Background

Implementation of best evidence is paramount to optimize post-stroke recovery outcomes [1, 2]. Clinical practice guidelines containing evidence-based recommendations have been proposed as a method to facilitate clinicians’ uptake of evidence [3–5]. A meta-review of 12 systematic reviews [6] categorized factors influencing guideline implementation into five main areas: 1) the guideline itself (e.g., guidelines that did not require specific resources were easier to implement); 2) the target health care professional user (e.g., less experienced health care professionals were more likely to implement guidelines than more experienced health care professionals); 3) patient characteristics (e.g., having patients with co-morbidities was associated with less guideline adherence by their health care professionals); 4) the work environment (e.g., limited resources and negative attitudes from colleagues lead to less clinical practice guideline adherence); and, 5) the type of implementation strategy used (e.g., a multifaceted intervention was shown to be more effective in implementing clinical practice guidelines than using one strategy only).

A variety of studies have demonstrated that stroke clinical practice guidelines are not routinely implemented [7–9]. For example, a 2005 Canadian study of 1800 stroke rehabilitation clinicians identified a significant gap between best and actual practices in stroke rehabilitation management. Specifically, there was a low prevalence of screening for high-risk, post-stroke sequelae and inconsistent use of assessment of important aspects of stroke recovery such as community reintegration and participation [7]. Complicating this scenario is the fact that stroke rehabilitation is characterized by an interdisciplinary team approach to care and the availability of multiple treatment recommendations. To date, there are no reports in the literature describing how to facilitate guideline implementation in this context.

The Canadian Stroke Network funded the Stroke Canada Optimization of Rehabilitation by Evidence (SCORE) Project team (Phase I). A consensus conference was held to address areas of stroke rehabilitation that require additional research. The priorities from this conference have been previously described [10]. In addition, our research team previously explored the facilitators and barriers to the implementation of the Evidence Informed Practice Recommendations in stroke to inform the KT interventions used in the intervention trial comparing the effectiveness of two KT interventions [11]. This approach is consistent with the finding that implementation strategies are more likely to be effective if they address local facilitators and barriers to change [12–14].

Phase II of the SCORE project was a cluster randomized implementation trial (SCORE-IT) that evaluated two KT interventions for the promotion of the uptake of best practice recommendations for interventions targeting upper extremity (UE) and lower extremity (LE) function, postural control, and mobility. Twenty rehabilitation centers across Canada were randomly assigned to either the facilitated or passive KT intervention (unpublished work). Facilitation is defined as “…enabling individuals, teams, and organizations to change”. There are many interpretations of the facilitator role in practice and they can involve a practical role of assisting change to a more complex, multi-dimensional role [15]. The specific details of the facilitated and passive KT intervention are presented in Table 1.

**Table 1:** Descriptions of the facilitated and passive knowledge translation interventions

	Facilitated knowledge translation intervention	Passive knowledge translation intervention
Personnel	There was funding for two facilitators (one nurse and one therapist).	None
Frequency and duration	4 h/week/facilitator in each intervention site to promote guideline implementation over a 16-month period	Not applicable
Components	At a two-day workshop: facilitators received change management education, a practice-change toolkit, information on successful guideline implementation strategies from the pilot study, slide presentations, and clinician-targeted media releases for marketing SCORE. They also completed training to apply treatments, compared current practice with recommended practice, identified barriers to practice change, developed a guideline implementation plan addressing barriers and incorporating behaviour change strategies, and learned how to conduct small group education/training sessions. In addition, stroke teams received SCORE guideline booklets with treatment recommendations and evidence-based treatment protocols, pocket reminder cards, and posters describing protocols designed for therapists or nurses. Teleconferences and a web-based platform were provided for facilitators to communicate and share successful strategies.	Sites in the passive KT intervention received a version of the SCORE guideline without treatment protocols, and a handbook and educational DVD on the use of standardized assessment tools post-stroke. In addition, clinicians were invited by email to participate in a list serve to obtain additional information or share experiences about the trial outcome measures.
Research team involvement	The research team provided external facilitation to the facilitators; specifically, advice and support via teleconferences.	None

Consistent with the Medical Research Council (MRC) Framework [16] for evaluating complex interventions, qualitative research is essential to understanding guideline implementation interventions (e.g., in this case, whether the KT interventions adequately addressed all of the barriers previously identified) and guide future efforts. There is a paucity of qualitative studies on the views of stakeholders and health care professionals regarding the implementation of stroke clinical guidelines and/or tools or interventions aimed to increase their uptake. However, a few qualitative studies exist on health care professionals’ perspectives on facilitators and barriers to stroke clinical guideline implementation. For example, Donnellan and colleagues [17], in qualitative study of perceived facilitators and barriers to implementing clinical guidelines in stroke (by stakeholders and health care professionals), determined that having dedicated resources, user-friendly guidelines relevant at the local level, and having supportive advocates acted as facilitators to implementation. Inadequate resources, poor guidelines characteristics, and insufficient training and education acted as barriers. Similarly, Miao and colleagues [18] examined factors affecting speech pathologists’ implementation of stroke management guidelines and determined that factors affecting implementation were complex and not exclusively facilitators or barriers. They identified the following three themes: making implementation explicit, demand versus ability to change, and motivation of speech pathologists to implement guidelines. To the best of our knowledge, no previous studies have examined the barriers and facilitators to the implementation of both the implementation of stroke clinical guidelines and the tools or interventions aimed to increase their uptake. Thus, the objective of the current study was to understand the facilitators and barriers influencing the implementation of the recommended treatments and KT interventions from the perspective of nurses, occupational therapists (OTs) and physical therapists (PTs), and clinical managers following completion of the SCORE-IT. We compared the identified facilitators and barriers influencing recommendation and KT intervention uptake by arm of the trial.

## Methods

### Guiding conceptual framework

The Clinical Practice Guidelines Framework for Improvement [19] and its updates [20–22] were used to guide the coding framework in the current study. Legaré and colleagues [21] formulated a definition for each type of barrier to promote the standardization in the reporting of barriers and facilitators across different studies. Examples of barriers and facilitators include knowledge (awareness, familiarity), attitudes (e.g., agreement with intervention), and behavior, including environmental factors [20–22]. The use of the Clinical Practice Guidelines Framework for Improvement [19] was not decided a priori. We decided to use the Clinical Practice Guidelines Framework for Improvement [19] and its updates [20–22] as it is one of the most recognized frameworks for assessing barriers and facilitators and after analyzing several focus groups and determining that the emerging categories clearly aligned with the barriers outlined in the framework.

### Design/Approach

This study took a qualitative descriptive approach that consisted of telephone focus groups. A qualitative descriptive approach is well-accepted for researching topics about which little is known and yielding practical answers of relevance to policy makers and health care practitioners [23, 24]. Telephone focus groups were selected because of the geographic dispersion (i.e., national scale) of the study participants.

### Recruitment

Participants included staff members from the three different professional groups, nurses, therapists (OTs and PTs), and health care/clinical managers, who had participated in the SCORE-IT and agreed to be contacted at the conclusion of the trial. Participants were contacted by telephone and email about their willingness to participate in the focus groups. Purposive sampling [25] was used to recruit equal numbers of participants across professional groups (nurse, therapist, clinical manager), randomization arms (facilitated KT intervention or passive KT intervention), and geographic locations (Western, Central, Quebec, Eastern). Uniprofessional focus groups were conducted for nurses, therapists, and clinical managers at each of the participating sites. This approach was adopted to mitigate potential power imbalances that may have influenced what participants might be willing to share. Participants were recruited between January 2009 and March 2010. Recruitment ceased when a discussion and review of the responses revealed that saturation had been achieved (i.e., no new responses or themes were emerging) [26].

### Data collection

Each participant took part in a semi-structured telephone focus group lasting approximately 45–60 min. The principal investigators (MB, SWD) and the research coordinators involved in the trial conducted the focus groups. The focus group guide consisted of semi-structured open-ended questions and was informed by the results of our pilot project [11]. The interview guide was pilot tested with a researcher experienced in qualitative methods. Probes or recursive questioning were used during the focus groups to explore issues in greater depth and to verify understanding of the information being collected [25]. The probes were revised and refined as data collection progressed to establish saturation [26]. The complete list of questions is included in Additional file 1. No repeat interviews were conducted. All focus groups were audio recorded. Field notes were made during and/or after the focus groups. The recordings were transcribed verbatim for data analysis. These transcripts were not returned to participants for comment and/or correction.

### Data analysis

To facilitate the organization and analysis of the qualitative data, the transcripts were entered into NVivo 10 [27]. Thematic analysis as described by Braun and Clark [28] was used to understand the factors influencing the implementation of the recommended treatments and KT interventions by study arms. The lead author (SM) reviewed the transcripts to develop an initial codebook based on the Clinical Practice Guidelines Framework for Improvement. Following this, two researchers (SM, MB) independently coded a sample of the transcripts (20%), revised the codebook as themes emerged, and met to discuss and reconcile discrepancies until agreement of the coded transcripts was reached. SM is a female post-doctoral fellow and has a PhD in Health Services Research as well as expertise in knowledge translation. She has approximately 10 years of experience conducting qualitative research. MB is a physiatrist (i.e., MD) with expertise in stroke, brain injury, rehabilitation, clinical practice guidelines, prognostic factors, and health services research. He has approximately 10 years of experience conducting qualitative research. Our background in knowledge translation science has influenced the conceptual frameworks that we have been exposed to including our knowledge and selection of the Clinical Practice Guidelines Framework for Improvement [19] for this study. Disagreements/discrepancies around codes, themes, and subthemes were resolved by discussion and reference to the original transcripts. The lead author (SM) analyzed the remaining transcripts. Relevant quotations were identified and selected from the transcripts to illustrate the themes and include the participant’s professional group (nurse, therapist, or clinical manager), and randomization arm (facilitated KT intervention or passive KT intervention). Participants were not provided feedback on the findings.

### Ethics and trial registration

Research ethics approval was obtained from each site and affiliated university. All participants provided written consent prior to the interview. The trial was registered at ClinicalTrials.gov (NCT00359593).

## Results

### Description of the rehabilitation centers

Focus groups were conducted with 33 individuals including 11 nurses, 11 therapists, and 11 clinical managers. There were between two and six participants in attendance at each focus group. Participants were from 11 of 20 sites in Western, Central and Eastern Canada as well as Quebec. This sample represented 6 sites from the facilitated KT arm and 5 sites from the passive KT arm.

### Overview of themes - facilitators and barriers influencing implementation of the SCORE-IT

Overall, five themes were identified. The following themes influencing the implementation of the recommended treatments and KT interventions emerged: facilitation, agreement with the intervention – practical, familiarity with the recommended treatments, and environmental factors (including time pressure, insufficient staff, lack of space and equipment, and organizational constraints). These themes, for the most part, emerged as facilitators and barriers influencing the implementation of the SCORE-IT. Furthermore, the theme of improved team communication and interdisciplinary collaboration emerged as an unintended outcome of the trial across both arms in addition to facilitating the implementation of the treatment recommendations. Representative quotes are given in Tables 2 and 3.

**Table 2:** Facilitators to Implementation

Theme	Quote	Source
Facilitation	Without the facilitator and the model, I don’t think we would have gotten as far as we did with the implementation of best practice.	Manager, Facilitated Site 4
	One of the physiotherapists initially oriented the staff as to what it was about, and I’ve always thought of her as the go-to person for information.	Nurse, Passive Site 3
	And what I tried to sort of make clear early on was that this, we were all in this project. Participation wasn’t an option so if either of the two sort of leaders of the project encountered any difficulties, be it with staff or lack of equipment, resources, anything like that, they were to let me know. Because if need be either [name] or I would have been the bad guy and stepped in. That never happened but they, I think they certainly felt that they weren’t out there on their own and we stressed at the beginning, it wasn’t their project, it was a [site name] project so they … We didn’t need to be the heavy but I think everyone understood that, you know, if need be we were there.	Manager, Facilitated Site 2
	I think [name] took a leadership role in regards to the project itself.	Therapist, Passive Site 5
Agreement with the Intervention – Practical	Yes, we had our pocket cards, little laminated pocket cards, and those were quite useful. And also the posters in the rooms, for positioning, if you weren’t sure it was right there, so that was wonderful.	Nurse, Facilitated Site 2
	The posters in the rooms were very helpful. The transfer and positioning posters were great to have above the beds, just to help make sure everything was done properly. The digital frame was also very helpful	Nurse, Facilitated Site 6
Familiarity with the Recommended Treatments	We were doing the CMSA (Chedoke-McMaster Stroke Assessment) before so that wasn’t new... We were already doing some of the upper extremity tasks…	Therapist, Passive Site 4
	From the recommendations, a lot of the stuff we were already doing. Things like aerobic conditioning, I found like the implementations reinforced that that was a good thing we were doing.	Manager, Passive Site 3
Team Communication and Interdisciplinary Collaboration	We have good relationships. Certainly it is a good relationship but I think it only got better when we did this particular education piece of it or how we particularly did it. Because I think we gave each of the groups a little more respect for the other group in terms of what they do. Because, you know, a lot of nurses really don’t know what OTs actually do because O therapy is a little more objective in terms of working with muscle groups and joints and things like this. But with OT, you know, they sort of take the patients off to the bathroom or up to their work area or whatever and they just don’t know a heck of a lot of what they do. But with this little educational piece that we did, they learned.	Nurse, Passive Site 1
	I think generally, among the physios, we tend to talk to each other a fair bit. And even among the OTs and the other team, if there’s issues, we’re talking. If not on a daily basis, then at least every 2 or 3 days. Definitely in rounds. Sort of even informally consulting in the corridors with stuff like that about various patients.	Therapist, Passive Site 1
	…we established early, early on was a committee, sort of a joint therapist, nursing staff, healthcare aid committee. So as we were moving forward communication happened within that committee.	Manager, Passive Site 2
Team Communication and Interdisciplinary Collaboration	The workshop was very good at explaining the “why” behind the treatment modalities, and I think that was helpful. That was more helpful than just a list of recommendations, because having a rationale and a justification for why a treatment is the best choice was useful.	Therapist, Passive Site 3
	Yes. From an interdisciplinary standpoint, PT and OT have a mixed office now. The stroke unit has an office now as well. There has been more collaboration and team work. The staff that has signed up to work on the dedicated stroke unit are working on the dedicated stroke unit. They interact more, and there is more collaboration	Manager, Facilitated Site 4
	And I think you’re also encouraging each other with it because now that you’ve gone through the education and been part of this project…	Nurse, Passive Site 1

**Table 3:** Barriers to implementation

Theme	Quote	Source
Lack of facilitation	It was good in theory, but we needed a person to continue with the program and reinforce it. After the booklets were handed out, we never went back to them, and I think the education needed to continue right away. If there had been someone whose main goal was to facilitate the implementation, without being pulled in different directions by other responsibilities, I think things would have gone much better.	Nurse, Facilitated Site 4
	We didn’t have somebody who I thought could be an actual overseer of this. If we were trying to do this again, it might be better to either have a senior being the person overseeing the project, or even get the nurse educator we have on board doing that kind of thing.	Manager, Passive Site 3
	We did have some new staff that came in and watched the DVD but had a bit of trouble with it because it was a very busy time for us so there was very little mentorship I think.	Therapist, Passive Site 3
	So I think one of the things I would suggest too is that we get a champion on the nursing unit to really, somebody who works on the unit. I mean I don’t work on the unit. I’m all over the building as an educator but somebody like [name] who would get specific education and be the champion, be the one that could be, you know, the supporter on the units, be encouraging the other staff that she’s working with to be involved and to be doing it. I think that would help…	Nurse, Passive Site 1
	We had one nurse who was more involved in SCORE, but because she didn’t work full time she wasn’t there every day so we missed a bit of the information and the teaching we could have gotten because she wasn’t a full time worker. I think we needed a full-time worker to be involved to have a more significant influence in pushing SCORE. She tried to get everyone aware of the project and the recommendations, and she transferred the information along to the staff. She made sure everyone was involved, and that everyone was up to date on the information about the project.	Nurse, Facilitated Site 6
Lack of agreement with the intervention – not practical	The DVD was a little dry. It was hard to stay awake during the presentation, so I really don’t remember much of it. At the time we were watching it we were short-staffed and trying to cram it into a lunch hour so it was hard to pay attention. I didn’t find the DVD all that useful.	Manager, Passive Site 3
	I know, for example that one of the recommendations for the frequency of the FES for the upper extremity was feasible for clients that were completely independent for the setup of the FES, but I think the two 30 min sessions per day recommendation was difficult to complete. Some of the recommendations were not so realistic to follow due to time constraints.	Therapist, Facilitated Site 6
Lack of familiarity with the recommended treatments	There were some knowledge barriers about the process of functional electrical stimulation. The specifics of being comfortable with doing it, and the intricacies, those were a barrier.	Manager, Passive Site 3
	I think the one thing I really struggled with before I left on Mat leave was starting the muscle stim just because I didn’t have the background as to why it was being used.	Therapist, Facilitated Site 2
	It could have been nice to see how that worked but we didn’t have the equipment or the education, and we wouldn’t have been comfortable doing FES without the proper training and knowledge.	Therapist, Facilitated Site 4
	I think we struggled the most with the [spell out acronym] CAHAI because that was new for a lot of us, and that we needed to review the most.	Therapist, Facilitated Site 7
Environmental factors [Lack of Space and Equipment]	Space was a bit of a barrier for the 6 min walk test, in terms of finding enough open space without obstacles.	Manager, Passive Site 5
	Equipment was also a barrier. Some of the slings seemed to go missing, and we didn’t have FES equipment so those are just a few examples.	Therapist, Facilitated Site 4
Environmental factors [Organizational Constraints]	It was a good experience, and I would do it again, but it was definitely a lot of work, and maybe it could have been more heightened with our leadership team.	Manager, Facilitated Site 4
	I would say no. They were aware of the project, and were given updates at the quarterly meetings, but they did not have a direct involvement.	Manager, Passive Site 5
Environmental factors [Time Pressure]	Another thing is I guess the implementation took more time than I thought it would, it was a bit harder to set things up than I expected.	Manager, Facilitated Site 4
	The CMSA was very time consuming, so I think people struggled with the time aspect, spending so much time on all of the implementations. The value of the activities was appreciated, but going through all of the tools and processes was time consuming and people resented how much time it took to implement everything.	Manager, Passive Site 3
	…we just haven’t got time. We’ve got new people coming in, we’ve got measures to record that we didn’t do before and I think an underlying problem is that the unit now is so busy that it isn’t adequately staffed for both physio and OT. And that’s a pre-existing problem. I think the study maybe just highlighted it a little bit but I think the therapists were, did feel a certain pressure because they knew they hadn’t got their assessments done. They knew they hadn’t got their discharge paperwork done but the patients just kept coming and coming and coming. And I’m sure that’s not unique to us and I’m sure, you know, if we gave them ten more therapists in a year’s time they’d say, they were short of staff. But I think the time was the big thing.	Manager, Facilitated Site 2
Environmental factors [Insufficient Staff]	We’ve been through a lot of change in the hospital, because our therapists rotate, so we were moved around a bit. That’s really part of the whole problem, because the staff that was up here got moved around, and the unit was closed for a bit around Christmas. It became difficult to incorporate best practices when we were dealing with all of these issues. I think we had to deal with therapists moving around and also being a bit understaffed.	Therapist, Passive Site 5
	We had staffing issues, especially OT staffing issues. We still have those issues. We’ve been at about 60% of OT staffing for a while now. The staffing for RN as well, we couldn’t get those ratios to the level we wanted.	Manager, Passive Site 4
	It was quite difficult since we were short staffed for probably two thirds of the duration of the project; it was difficult to do new things. When we hired a senior therapist she came on board for the last 3 months and she was able to start implementing things we would have liked to have done, some of the recommendations. I think if you have the right people, it is much easier but when we were short staffed that limited us and we just tried to do our best.	Manager, Passive Site 3
Environmental factors [Lack of Space and Equipment]	Space was a bit of a barrier for the 6 min walk test, in terms of finding enough open space without obstacles.	Manager, Passive Site 5
	Equipment was also a barrier. Some of the slings seemed to go missing, and we didn’t have FES equipment so those are just a few examples.	Therapist, Facilitated Site 4
Environmental factors [Organizational Constraints]	It was a good experience, and I would do it again, but it was definitely a lot of work, and maybe it could have been more heightened with our leadership team.	Manager, Facilitated Site 4
	I would say no. They were aware of the project, and were given updates at the quarterly meetings, but they did not have a direct involvement.	Manager, Passive Site 5
Lack of team communication and interdisciplinary collaboration	Well I haven’t even seen these recommendations, so I think getting together as a group and discussing it would have been useful. We should have had a meeting together to go over it, because I think we only really knew about the sheets where we ticked off the patients’ progress.	Nurse, Passive Site 5
	Some of the feedback I heard was that it would have been great if we had a blog or if we had a bulletin board of some sort that people would go in, post their question, had their questions answered and that kind of thing. So it would have been a little more timely in terms of getting things off the ground and that kind of stuff. It would have provided more timely clarification I think.	Manager, Passive Site 1
	I would, like I was saying, just maybe more communication between the disciplines as to what’s going on. That nurse facilitators may be having a meeting every few months or whatever it is, just to make sure that everybody is on the same page.	Nurse, Passive Site 2

### Facilitation

#### Facilitator

Facilitation often involved individuals who championed the trial and its recommendations and/or interventions. The majority of participants representing all professional groups and randomization arms noted that facilitation enhanced recommendation and KT intervention uptake. In the facilitated KT intervention sites, the theme of facilitation often referred to the designated facilitators in this arm of the trial (i.e., having two facilitators 4 h/week/facilitator). In the passive KT sites, facilitation was usually self-initiated by a local staff member (frequently a manager) who appeared to be highly motivated. For example, informal workshops or team activities were initiated by such individuals at some passive KT intervention sites. Participants in both arms of the trial indicated that staff acting as facilitators provided support and motivation to their colleagues. Furthermore, the presence of the facilitator often provided continuity for the trial (procedures/tasks) in the face of high staff turnover.

#### Barrier

A lack of facilitation (i.e., not enabling individuals, teams, and organizations to change) was a barrier identified by all the professional groups and by those in the passive KT arm, in particular, as hindering the uptake of the KT interventions. This theme involved the lack of an individual(s) to champion the trial. A lack of facilitation had implications for mentorship of other staff members, the continuity of the project overall (especially in the face of staff turnover), and the sustainability of the KT interventions during the trial period and beyond (e.g., to champion the use of the DVD).

### Agreement with the intervention – practical

#### Facilitator

According to Legaré and colleagues [21, 22], the definition of agreement with the intervention – practical is the following: “…agreement with [an intervention] because it is clear or practical to follow”. Nurses and therapists, in the facilitated KT arm stated that elements of the KT intervention, including the posters in patient rooms for positioning for shoulder pain prevention, were clear and practical to follow, and supported implementation of the recommendations. Specifically, nurses and therapists noted that the posters were specific and could be used at the point of care. As such, they were regarded favorably (i.e., judged to increase the quality of care) and were frequently used.

#### Barrier

According to Legaré and colleagues [21, 22], the definition of lack of agreement with the intervention – i.e., not practical is the following: “lack of agreement with [an intervention] because it is unclear or impractical to follow”. In contrast, across both arms of the trial, participants indicated that components of the trial that were reportedly unclear and/or not practical served as a barrier to implementation. The KT interventions that were discussed most frequently as not practical were watching the DVD (passive KT arm) and the pocket cards (facilitated KT arm). For example, staff in the facilitated KT arm indicated that they have too many pocket cards and that the information was too general. Across both arms of the trial, participants also mentioned that certain recommendations/tests were not practical to implement because they were time-consuming (e.g., functional electrical stimulation (FES unit)).

### Familiarity with the recommended treatments

#### Facilitator

PTs and OTs, in both trial arms indicated that having some recommendations already in use at the site served to encourage their wider uptake. Aerobic conditioning and some of the positioning practices were the most commonly cited recommendations already being used in practice. Many of the participants indicated that the inclusion of the evidence-based recommendations in the trial underscored their importance.

#### Barrier

Participants across the professionals groups and across both arms of the trial indicated that a lack of familiarity with the recommended treatments, including equipment (e.g., FES unit) and assessment tools (e.g., Chedoke Arm and Hand Inventory (CAHAI)), discouraged implementation efforts. Participants also noted that a lower volume of patients, which was associated with fewer opportunities to become familiar with the tools/measures and/or equipment, limited their ability to implement certain components of the trial.

### Environmental factors

#### Barrier

Almost all of the participants in both arms of the trial indicated that time pressure was a key obstacle to implementation of the KT interventions. This barrier often coincided with a lack of staff or staff turnover (i.e., insufficient staff) and was related to a lack of funding for additional positions. Time pressure was also associated with competing initiatives and/or roles/responsibilities of staff members. In addition, participants in both arms indicated that some of the recommended treatments/measures themselves were time-consuming to implement. Barriers associated with the environment also included a lack of space and equipment needed to perform the recommendations (e.g., the 6 min walk test, the lack of a FES unit). Finally, some of the participants noted a lack of active support from senior management for the implementation of the trial despite their senior management sanctioning the project.

### Team communication and interdisciplinary collaboration

#### Facilitator

Across both arms of the trial, managers in particular noted that increased team communication and interdisciplinary collaboration were facilitators to the implementation of the recommended treatments (fostered via the educational interventions in both arms of the trial).

#### Unintended outcome

At other sites, it was noted that the KT interventions had the unintended benefit of increasing team communication and interdisciplinary collaboration (via the educational sessions or in discussing the DVD). This was also noted across both arms of the trial. In particular, some participants noted that the collaboration between PTs and OTs improved as a result of the trial. A greater understanding of the roles and responsibilities of each professional group was also noted, particularly for the roles and responsibilities of OTs.

## Discussion

### Summary of main findings

The objective of the current study was to understand the facilitators and barriers influencing the implementation of the recommended treatments and KT interventions from the perspective of nurses, OTs and PTs, and clinical managers following completion of the SCORE-IT. This is one of the first studies to examine the factors influencing the implementation of evidence-informed stroke recommendations and associated KT interventions among allied health care professionals within the context of a trial.

All of the factors influencing the implementation of the recommended treatments and KT interventions including facilitation, agreement with the intervention – practical, familiarity with the recommended treatments, environmental factors, and team communication and interdisciplinary collaboration were identified in both arms of the trial. Team communication and interdisciplinary collaboration also emerged as an unintended outcome of the trial in both arms of the trial. It is particularly noteworthy that facilitation was identified as a facilitator to implementation in the passive KT intervention arm despite the lack of formally instituted facilitators in this arm of the trial. The order of the remainder of this Discussion section is the same as the Results section. As in the Results section, where applicable, we have used the same terms from the Clinical Practice Guidelines Framework for Improvement [19] as headings to organize our Discussion section.

### Role of facilitation

The presence or absence of facilitation in this trial emerged as both a facilitator and barrier to implementation of the recommendations and KT interventions. Indeed, the results of a systematic review on local opinion leaders and their effects on professional practices revealed that opinion leaders alone or in combination with other interventions may successfully promote evidence-based practice [29]. In fact, research on barriers to research use in health care have consistently identified the behaviors of managers and their lack of leadership as major limiting factors to research use by clinicians [30–34].

One of the main findings of this study was that the theme of facilitation was noted in both arms of the trial, despite the fact that only the facilitated KT arm had formal facilitators. Previous research has raised the question of whether the process by which opinion leaders are selected affects the success of educational initiatives [29]. If self-selected facilitators (i.e., in this case, staff at the passive KT sites) are more beneficial (to implementation outcomes) than facilitators who are selected by external influences, it is possible that other components of the facilitated KT arm of the trial may not have been optimized. Alternatively, the self-initiated facilitation roles taken by staff members at the rehabilitation centers in the passive KT arm may explain why the outcomes at these centers were better than anticipated. Indeed, the results of the trial revealed that while the facilitated KT intervention was associated with a significantly greater improvement in the rate of implementing sit-to-stand training and walking practice, the passive KT intervention was associated with significantly greater improvement in the rate of implementing standing balance training (after adjusting for clustering at patient and provider levels and covariates) (i.e., between group differences) (unpublished work). It should be noted that the original trial was dealt with as a pragmatic trial, which tries to mimic the usual care situation and not impose too many fidelity standards on the basis that they produce a trial result which is not applicable/externally valid for the use of the same intervention under usual care conditions. Furthermore, as Horne [35] noted, staff can be trained to be good managers, but leadership is less susceptible to training and is better obtained by selective recruitment. This phenomenon may explain why facilitation (including a lack of facilitation) was noted across both arms of the trial. At the same time, we are not linking facilitation behaviour to the actual use of the recommendations, rather, we are presenting perceptions of what may or may not have occurred (i.e., in the control arm, it appears that individuals in some sites stepped up to try to mobilize and encourage the uptake of recommendations and the KT interventions themselves). Future research should seek to better understand the specific characteristics/behaviours of facilitators that are associated with successful implementation and clinical outcomes, especially within the context of stroke rehabilitation.

### Role of practicality/familiarity

Practicality of and familiarity with the recommended treatments and KT interventions also emerged as significant facilitators and barriers. For example, participants indicated that certain recommendations/tests were not practical to implement because they were time-consuming (e.g., FES unit). Indeed, it is likely that a variety of facilitators and barriers acted together and in combination to influence the implementation of the interventions in the trial (e.g., interaction of time and practicality). Similarly, a lack of familiarity with certain components of the recommended treatments, including equipment such as the FES apparatus and measures such as the CAHAI, limited the implementation efforts. Indeed, results from the SCORE-IT indicated that complex treatments that either involved multiple steps or technology, including the FES, were rarely implemented at baseline and demonstrated either no change or reduced application post-intervention (unpublished work). This finding suggests that the KT interventions did not adequately overcome these barriers. It is possible that these barriers cannot be overcome with KT interventions, especially within the context of a trial (i.e., lack of familiarity with a recommendation(s) and its implementation can only be overcome with a significant amount of time); however, it is possible that facilitation (i.e., mentorship) could be leveraged to overcome barriers associated with practicality and/or familiarity. Furthermore, previous research has reported that insufficient skills and a lack of experience with guideline recommendations are key barriers to implementation of best practices [6, 11, 36–38]. It could be that a mid-point check of progress and renewed goal setting might be helpful to address these barriers.

### Role of environmental factors

Environmental factors, including time pressure, insufficient staff (lack of staff, staff turnover), lack of space and equipment, and organizational constraints (insufficient support from the organizational/senior management) emerged as the most frequently cited barriers to implementation of the KT interventions during the trial as well as the recommendations. In a recent study describing the factors influencing the implementation of stroke clinical practice guidelines among speech pathologists, Hadely and colleagues [36] also reported that factors within the work environment were barriers to implementation. Specifically, the main barriers included lack of time, education, treatment resources, and standardized assessments to carry out guideline implementation [36]. Environmental/work factors as barriers (and facilitators) to guideline implementation have been reported consistently in literature – in the treatment of persons post-stroke as well as other chronic conditions [6, 11, 37, 39, 40]. In the current study, one of the main findings was that environmental factors were seldom noted as facilitators to the implementation of the recommended treatments and/or KT interventions. It should also be highlighted that some of these environmental factors were mitigated by team factors/facilitation. Thus, a main message from our research is that in the absence of more organizational resources (time, money), team factors can be leveraged to overcome such deficits. For example, a high level of staff turnover was noted across the rehabilitation centers; however, if strong leadership/management support was present at the rehabilitation center, this person often ensured that new staff knew the procedures/responsibilities associated with the trial. Horne [35] similarly noted that leaders and even the larger hospital administrative culture could act as key mediators between the environmental factors (time, money, equipment) and the implementation of recommendations.

### Role of team communication and interdisciplinary collaboration

The presence of team factors, including communication and interdisciplinary collaboration, served as a facilitator to the implementation of the recommended treatments. Donnellan and colleagues [17] also determined that barriers to adherence to generic stroke guidelines related to organization and multidisciplinary team factors [41–43]. Similarly, team factors played a significant role in influencing the implementation of stroke clinical practice guidelines in the study by Hadely and colleagues [36]. For example, they determined that working in a multi-disciplinary team emerged as a main factor for facilitating the use of guidelines among speech pathologists. These factors have also been reported among physicians, nurses, and OTs [11, 40, 44–47]. Hadely and colleagues [36] concluded that fostering teamwork can have a significant influence not only in improving guideline implementation but also patient functional gains [48] and length of hospital stay [49].

### Use of the clinical practice guidelines framework for improvement

Findings from the current study also suggest that the Clinical Practice Guidelines Framework for Improvement [19] is relevant in the context of implementing recommended treatments and KT interventions in stroke rehabilitation as agreement with the intervention – practical, familiarity (with the recommended treatments), and three aspects of environmental factors were identified as factors influencing implementation. The other identified factors of facilitation and team communication and interdisciplinary collaboration are not included in the Clinical Practice Guidelines Framework for Improvement [19] and its updates [20–22] but are included in other implementation frameworks, namely the Consolidated Framework for Implementation Research (i.e., formally appointed internal implementation leaders versus champions, networks and communications) [50] and the Promoting Action on Research Implementation in Health Services (PARIHS) (i.e., facilitation) [51]. Future iterations of the Clinical Practice Guidelines Framework for Improvement [19] could consider these factors, which may improve its ability to address common facilitators and barriers in this context. Lastly, another area of future research would be determining the perceived relative importance of these identified facilitators and barriers (e.g., using a modified Delphi process).

### Comparison of identified facilitators and barriers to pilot project

It is noteworthy that many of the identified factors influencing the implementation of the recommended treatments and KT interventions were also identified in our previous multi-site pilot project on the barriers to the implementation of evidence-based recommendation for stroke rehabilitation (i.e., lack of time, inadequate staffing, and equipment). As previously identified, many of these environmental barriers are difficult to overcome and beyond the control of the trial implementation effort. We also previously noted that leaders at the organizational level may be required to overcome these issues; however, in the current study, organizational constraint (insufficient support from the organizational/senior management) was a noted barrier across both arms of the trial. Thus, we may not have adequately addressed these barriers (and the interrelated nature of these barriers) in the current trial. At the same, team functioning and communication was previously noted in the pilot study but was identified as both a facilitator and unintended benefit in the current study.

### Limitations

We acknowledge some limitations. Only one person coded the majority of the data, which may have resulted in bias. The persons conducting the original study also conducted the focus groups, which presents a significant concern about the social desirability of participant responses. At the same time, however, the focus group leaders were careful to avoid biasing the participants towards or away from the intervention and were themselves neutral on its effectiveness (i.e., clinical equipoise). Furthermore, only 11 of the 20 sites participated (but almost equal representation from the facilitated KT and passive KT arms), and thus it is possible that a selection bias operated in that those participants who agreed to take part in this study may have had a greater interest and success with implementing evidence-based recommendations for stroke than those individuals who chose not to participate (i.e., limiting the applicability of the study findings). In discussing factors influencing the implementation of the KT interventions following completion of the trial, participants may have had recall bias; in a focus group setting, participants may also have felt limited in their ability to share their experiences due to social desirability issues. Organizing the focus groups by professional group was an attempt to mitigate this potential barrier. Furthermore, the focus group questions did not specifically ask about all of the factors influencing the 18 recommended treatments of interest (e.g., training for sitting balance, training for standing balance) or did not consistently ask about each of the KT interventions. As such, we are only able to obtain a global sense of the factors influencing implementation of the recommended treatments and their associated KT interventions. This may mask specific issues for specific interventions. The focus group questions were not anchored on the actual performance of the rehabilitation centres; more specific knowledge about the facilitators and barriers to implementation would have been obtained if this approach has been adopted (as discussed above in the Role of Facilitation section). Lastly, the trial and the subsequent focus groups were conducted a number of years ago; it is unknown how a more recent implementation of stroke recommendations and interventions to increase their uptake would affect the current results (e.g., with health system advances such as electronic medical records with reminders).

## Conclusions

Factors influencing the implementation of the recommended treatments and KT interventions including facilitation, agreement with the intervention – practical, familiarity with the recommended treatments, environmental factors, and team communication and interdisciplinary collaboration were identified in both arms of the trial. Despite the absence of formally instituted facilitators in the passive KT arm, facilitation was identified as an important facilitator influencing implementation of the KT interventions in this arm of the trial. This may suggest the important role of self-selected facilitators to implementation efforts. Future research should seek to better understand the specific characteristics/behaviours of facilitators that are associated with successful implementation and clinical outcomes, especially within the context of stroke rehabilitation. Lastly, the current study highlights the challenges of overcoming environmental factors including time pressures and insufficient staff in implementation efforts and the need for organizational support to mitigate these challenges.

## Additional file

Additional file 1: SCORE-IT Interview Guide for Facilitated and Active Sites’ Focus Groups. Description of data: Interview guides for facilitated and active sites’ focus groups including guides for the clinical managers, therapists, and nurses. (DOCX 40 kb)

## Abbreviations

CAHAI: Chedoke Arm and Hand Inventory
EIPR: Evidence Informed Practice Recommendation
FES: Functional electrical stimulation
OT: Occupational therapist
PT: Physical therapists
SCORE-IT: Stroke Canada Optimization of Rehabilitation by Evidence Implementation Trial
