# Supplementary material for: Perspectives of health care professionals on the facilitators and barriers to the implementation of a stroke rehabilitation guidelines cluster randomized controlled trial
Source: BMC Health Serv Res. 2017 Jun 26;17:440. doi: 10.1186/s12913-017-2389-7 (PMC5485614; doi:10.1186/s12913-017-2389-7)
Supplement: Additional file 1: — SCORE-IT Interview Guide for Facilitated and Active Sites’ Focus Groups. Description of data: Interview guides for facilitated and active sites’ focus groups including guides for the clinical managers, therapists, and nurses. (DOCX 40 kb) [file 12913_2017_2389_MOESM1_ESM.docx]

**SCORE-IT Interview Guide for Facilitated Sites’ Focus Groups**

**A. Site Collaborator and Management Interview**

1. **Introduction:**

Over the past 18 to 20 months you have been involved in the SCORE-IT project. Today I am here to get your feedback about your experience of participating in the SCORE implementation project. Your feedback is very important. We will use the results to implement evidence based stroke rehabilitation across Canada.

1. **Ground Rules**
   - No specific names of patients, coworkers, or hospital administrative personnel should be used.
   - Confidentiality: What we say in terms of specific people and their comments stays in this room. You should understand that your comments will be kept confidential by the project team as well.
   - Consent form: Each of you signed a consent form at the beginning of the research project agreeing that we can audio-record this session and that we can use the information and that your name is confidential and will never be used in any publications or presentations of this work. Do you still agree to this?

In addition, we would like to stress that you have been invited here to share your thoughts. There are no right or wrong answers. I want you to try and be as open and honest as you can be. We will start by discussing your personal experience with SCORE-IT and then we can discuss your overall impressions of implementing EBRs.

1. **Interview Questions:**

**Opening Question:** Let’s start by hearing from each of you about what happened during the SCORE-IT project.

Now we have some specific questions:

***Extent to which the proposed interventions were implemented:***

1. Tell me about your experiences implementing specific SCORE recommendations.

*Probes: What practices have changed?*

- - *Sit-to-stand*
  - *FES*
  - *Tasks for upper extremity*
  - *Intensive therapy (2 hours or more)*
  - *Aerobic exercises*
  - *Position of shoulder and shoulder pain*

1. Were there recommendations that your site did not implement?

*Probes:* *Can you give us examples? Explain why?*

***Process of implementation at site:***

1. Did the implementation of the recommendations necessitate a significant change in clinician practice? If so, can you provide examples?
2. Did the recommendations change the way you and your team work (if at all)? If so, please describe.
3. Did any individual(s) assume a leadership or coordinating role of the implementation at your site? If so, can you describe what was done to help the implementation?
4. How was your hospital’s senior management (i.e., CEO, VP, Director General) involved in this project? Did they provide any support or assistance in any way?

***Barriers or facilitators to implementation:***

1. Describe any barriers in your practice environment to implementing the strategy at your site. *Probes:*
   - - *Presence of other studies*
     - *Time*
     - *Equipment*
     - *Communication*
     - *Space*
     - *Lack of support*
2. Tell me about your experience as site collaborator.

*Probe:* What were some challenges? How did you deal with these challenges?

1. In your opinion, how successful was the process oriented strategy used by the SCORE team at your site in assisting you to implement the SCORE recommendations?

*Probe: Any feedback regarding the following?*

- - *Therapy facilitator*
  - *Nursing facilitator*
  - *Gap analysis*
  - *Protocol*
  - *Pocket cards*
  - *PowerPoint presentations*
  - *Posters*
  - *Marketing articles*

***Feedback for improvement:***

1. Describe the feedback and questions you received from the providers regarding SCORE-IT and the EBRs. Based on the provider feedback, how do you think the process could have been improved?
2. Any other final thoughts you would like to share with us?

We have a quick checklist that we would like you to complete and return.

We would like to thank you for your participation in today’s focus group and as well for all of your hard work throughout the past few months. We appreciate all the valuable time you have taken to participate in the SCORE-IT. We will summarize the thoughts and comments you have provided to explain the main findings of the study.

**B. Facilitator Interview**

1. **Introduction**

Over the past 18 to 20 months you have been involved in the SCORE-IT project. Today I am here to get your feedback about your experience of participating in the SCORE implementation project. Your feedback is very important. We will use the results to implement evidence based stroke rehabilitation across Canada.

1. **Ground Rules**
   - No specific names of patients, coworkers, or hospital administrative personnel should be used.
   - Confidentiality: What we say in terms of specific people and their comments stays in this room. You should understand that your comments will be kept confidential by the project team as well.
   - Consent form: Each of you signed a consent form at the beginning of the research project agreeing that we can audio-record this session and that we can use the information and that your name is confidential and will never be used in any publications or presentations of this work. Do you still agree to this?

In addition, we would like to stress that you have been invited here to share your thoughts. There are no right or wrong answers. I want you to try and be as open and honest as you can be. We will start by discussing your personal experience with SCORE-IT and then we can discuss your overall impressions of implementing EBRs.

1. **Interview Questions:**

**Opening Question:** Let’s start by hearing from each of you about what happened during the SCORE-IT project.

Now we have some specific questions:

***Facilitator workshop and facilitator communication:***

1. How did the workshop prepare you for your role as facilitator?

*Probes: How was the workshop most helpful?*

*How could we improve the workshop to prepare facilitators better?*

1. Did the barriers/gap analysis that you developed at the workshop help to guide your implementation plan? Please explain.
2. Did you find communicating with the other facilitators (at other sites) helpful? Please explain your answer.

*Probes: Teleconferences*

*Website*

***Barriers or facilitators to implementation:***

1. Tell me about your experience as a facilitator?
2. Describe any barriers in your practice environment to implementing the strategy at your site.

*Probes:*

- *Presence of other studies*
  - *Time*
  - *Equipment*
  - *Communication*
  - *Space*
  - *Lack of support*

*How did you deal with these barriers?*

*What changes in your work environment would make it easier to use the recommendations?*

***Extent to which the proposed interventions were implemented:***

1. Tell me about your experiences implementing specific SCORE recommendations.

*Probes: What practices have changed?*

- - *Sit-to-stand*
  - *FES*
  - *Tasks for upper extremity*
  - *Intensive therapy (2 hours or more)*
  - *Aerobic exercises*
  - *Position of shoulder and shoulder pain*

*Which recommendations were easy to use in practice and why?*

1. Were there recommendations that your team did not implement? Can you give us examples and explain why?

*Probes: Which recommendations were difficult to use? Why?*

*Were there recommendations appropriate for a given patient that your team chose not to use? How did you make this decision?*

***Process of implementation at site:***

1. Did the implementation of the recommendations necessitate a significant change in practice? If so, can you provide examples?

*Probe: How did the recommendations change the way you and your team work (if at all)?*

1. In your opinion, how successful was the process oriented strategy used by the SCORE team at your site in assisting you to implement the SCORE recommendations?

*Probe: Any feedback regarding the following?*

- - *Therapy facilitator*
  - *Nursing facilitator*
  - *Gap analysis*
  - *Protocol*
  - *Pocket cards*
  - *PowerPoint presentations*
  - *Posters*
  - *Marketing articles*

1. How was your hospital’s senior management (i.e., CEO, VP, Director General) involved in this project? Did they provide any support or assistance in any way?

**Feedback for improvement:**

1. Describe the feedback and questions you received from the providers regarding the recommendations. Based on the provider feedback, how do you think the clinical protocol and pocket cards could have been improved?
2. Any other final thoughts you would like to share with us?

We have a quick checklist that we would like you to complete and return.

We would like to thank you for your participation in today’s focus group and as well for all of your hard work throughout the past few months. We appreciate all the valuable time you have taken to participate in the SCORE-IT. We will summarize the thoughts and comments you have provided to explain the main findings of the study.

**C. Therapists Interview**

1. **Introduction**

Over the past 18 to 20 months you have been involved in the SCORE-IT project. Today I am here to get your feedback about your experience of participating in the SCORE implementation project. Your feedback is very important. We will use the results to implement evidence based stroke rehabilitation across Canada.

1. **Ground Rules**
   - No specific names of patients, coworkers, or hospital administrative personnel should be used.
   - Confidentiality: What we say in terms of specific people and their comments stays in this room. You should understand that your comments will be kept confidential by the project team as well.
   - Consent form: Each of you signed a consent form at the beginning of the research project agreeing that we can audio-record this session and that we can use the information and that your name is confidential and will never be used in any publications or presentations of this work. Do you still agree to this?

In addition, we would like to stress that you have been invited here to share your thoughts. There are no right or wrong answers. We want you to try and be as open and honest as you can be. We will start by discussing your personal experience with SCORE-IT and then we can discuss your overall impressions of implementing EBRs.

1. **Interview Questions:**

**Opening Question:** Let’s start by hearing from each of you about what happened during the SCORE-IT project.

Now we have some specific questions:

***Extent to which the proposed interventions were implemented:***

1. Tell me about your experiences implementing specific SCORE recommendations.

*Probes: What practices have changed?*

- - *Sit-to-stand*
  - *FES*
  - *Tasks for upper extremity*
  - *Intensive therapy (2 hours or more)*
  - *Aerobic exercises*
  - *Position of shoulder and shoulder pain*

*Which recommendations were easy to use in practice and why?*

1. Were there recommendations that your site did not implement? Can you give us examples and explain why?

*Probes:* *Which recommendations were difficult to use? Why?*

*Were there recommendations appropriate for a given patient that your team chose not to use? How did you make this decision?*

**Process of implementation at site:**

1. Did the implementation of the recommendations necessitate a significant change in practice? If so, can you provide examples?

*Probe: How did the recommendations change the way you and your team work (if at all)?*

1. Did any individual(s) assume a leadership or coordinating role of the implementation at your site? If so, can you describe what that person did to help the implementation?

**Barriers or facilitators to implementation:**

1. Describe any barriers in your practice environment to implementing the strategy at your site. *Probes:*
   - - *Presence of other studies*
     - *Time*
     - *Equipment*
     - *Communication*
     - *Space*
     - *Lack of support*

*What changes in your work environment would make it easier for you to use the recommendations that you found challenging to implement?*

1. In your opinion, how successful was the process oriented strategy used by the SCORE team at your site in assisting you to implement the SCORE recommendations?

*Probe: Any feedback regarding the following?*

- - *Therapy facilitator*
  - *Nursing facilitator*
  - *Gap analysis*
  - *Protocol*
  - *Pocket cards*
  - *PowerPoint presentations*
  - *Posters*
  - *Marketing articles*

**Feedback for Improvement:**

1. What advice would you have for another centre thinking of implementing the SCORE recommendations or other clinical guidelines?
2. Any other final thoughts you would like to share with us?

We have a quick checklist that we would like you to complete and return.

We would like to thank you for your participation in today’s focus group and as well for all of your hard work throughout the past few months. We appreciate all the valuable time you have taken to participate in the SCORE-IT. We will summarize the thoughts and comments you have provided to explain the main findings of the study.

**D. Nurses Interview**

1. **Introduction**

Over the past 18 to 20 months you have been involved in the SCORE-IT project. Today I am here to get your feedback about your experience of participating in the SCORE implementation project. Your feedback is very important. We will use the results to implement evidence based stroke rehabilitation across Canada.

1. **Ground Rules**
   - No specific names of patients, coworkers, or hospital administrative personnel should be used.
   - Confidentiality: What we say in terms of specific people and their comments stays in this room. You should understand that your comments will be kept confidential by the project team as well.
   - Consent form: Each of you signed a consent form at the beginning of the research project agreeing that we can audio-record this session and that we can use the information and that your name is confidential and will never be used in any publications or presentations of this work. Do you still agree to this?

In addition, we would like to stress that you have been invited here to share your thoughts. There are no right or wrong answers. I want you to try and be as open and honest as you can be. We will start by discussing your personal experience with SCORE-IT and then we can discuss your overall impressions of implementing EBRs.

1. **Interview Questions:**

**Opening Question:** Let’s start by hearing from each of you about what happened during the SCORE-IT project.

Now we have some specific questions:

***Extent to which the proposed interventions were implemented:***

1. Tell me about your experiences implementing specific SCORE recommendations.

*Probes: What practices have changed?*

- - *Sit-to-stand*
  - *FES*
  - *Tasks for upper extremity*
  - *Intensive therapy (2 hours or more)*
  - *Aerobic exercises*
  - *Position of shoulder and shoulder pain*

*Which recommendations were easy to use in practice and why?*

1. Were there recommendations that your site did not implement? Can you give us examples and explain why?

*Probes:* *Which recommendations were difficult to use? Why?*

*Were there recommendations appropriate for a given patient that your team chose not to use? How did you make this decision?*

***Process of implementation at site:***

1. Did the implementation of the recommendations necessitate a significant change in practice? If so, can you provide examples?

*Probe: How did the recommendations change the way you and your team work (if at all)?*

1. Did any individual assume a leadership or coordinating role of the implementation at your site? If so, can you describe what that person did to help the implementation?

***Barriers or facilitators to implementation:***

1. Describe any barriers in your practice environment to implementing the strategy at your site. *Probes:*
   - - *Presence of other studies*
     - *Time*
     - *Equipment*
     - *Communication*
     - *Space*
     - *Lack of support*

*What changes in your work environment would make it easier for you to use the recommendations that you found challenging to implement?*

1. In your opinion, how successful was the process oriented strategy used by the SCORE team at your site in assisting you to implement the SCORE recommendations?

*Probe: Any feedback regarding the following?*

- - *Therapy facilitator*
  - *Nursing facilitator*
  - *Gap analysis*
  - *Protocol*
  - *Pocket cards*
  - *PowerPoint presentations*
  - *Posters*
  - *Marketing articles*

***Feedback for improvement:***

1. What advice would you have for another centre thinking of implementing the SCORE recommendations or other clinical guidelines?
2. Any other final thoughts you would like to share with us?

We have a quick checklist that we would like you to complete and return.

We would like to thank you for your participation in today’s focus group and as well for all of your hard work throughout the past few months. We appreciate all the valuable time you have taken to participate in the SCORE-IT. We will summarize the thoughts and comments you have provided to explain the main findings of the study.

**SCORE-IT Interview Guide for Passive Sites’ Focus Groups**

**A. Site Collaborator and Management Interview**

1. **Introduction:**

Over the past 18 to 20 months you have been involved in the SCORE-IT project. Today I am here to get your feedback about your experience of participating in the SCORE implementation project. Your feedback is very important. We will use the results to implement evidence based stroke rehabilitation across Canada.

1. **Ground Rules**
   - No specific names of patients, coworkers, or hospital administrative personnel should be used.
   - Confidentiality: What we say in terms of specific people and their comments stays in this room. You should understand that your comments will be kept confidential by the project team as well.
   - Consent form: Each of you signed a consent form at the beginning of the research project agreeing that we can audio-record this session and that we can use the information and that your name is confidential and will never be used in any publications or presentations of this work. Do you still agree to this?

In addition, we would like to stress that you have been invited here to share your thoughts. There are no right or wrong answers. I want you to try and be as open and honest as you can be. We will start by discussing your personal experience with SCORE-IT and then we can discuss your overall impressions of implementing EBRs.

1. **Interview Questions:**

**Opening Question:** Let’s start by hearing from each of you about what happened during the SCORE-IT project.

Now we have some specific questions:

***Extent to which the proposed interventions were implemented:***

1. Tell me about your experiences implementing specific SCORE recommendations.

*Probes: What practices have changed?*

- - *Sit-to-stand*
  - *FES*
  - *Tasks for upper extremity*
  - *Intensive therapy (2 hours or more)*
  - *Aerobic exercises*
  - *Position of shoulder and shoulder pain*

1. Were there recommendations that your site did not implement?

*Probes:* *Can you give us examples? Explain why?*

***Process of implementation at site:***

1. Did the implementation of the recommendations necessitate a significant change in clinician practice? If so, can you provide examples?
2. Did the recommendations change the way you and your team work (if at all)? If so, please describe.
3. Did any individual(s) assume a leadership or coordinating role of the implementation at your site? If so, can you describe what was done to help the implementation?
4. How was your hospital’s senior management (i.e., CEO, VP, Director General) involved in this project? Did they provide any support or assistance in any way?

***Barriers or facilitators to implementation:***

1. Describe any barriers in your practice environment to implementing the strategy at your site. *Probes:*
   - - *Presence of other studies*
     - *Time*
     - *Equipment*
     - *Communication*
     - *Space*
     - *Lack of support*
2. Tell me about your experience as site collaborator.

*Probe:* What were some challenges? How did you deal with these challenges?

1. In your opinion, how successful was the outcomes oriented strategy used by the SCORE team at your site in assisting you to implement the SCORE recommendations?

*Probes: Any feedback regarding the following?*

- 1. *DVD presentation*
  2. *Recommendations*

***Feedback for improvement:***

1. Describe the feedback and questions you received from the providers regarding SCORE-IT and the EBRs. Based on the provider feedback, how do you think the process could have been improved?
2. Any other final thoughts you would like to share with us?

We have a quick checklist that we would like you to complete and return.

We would like to thank you for your participation in today’s focus group and as well for all of your hard work throughout the past few months. We appreciate all the valuable time you have taken to participate in the SCORE-IT. We will summarize the thoughts and comments you have provided to explain the main findings of the study.

**B. Therapists Interview**

1. **Introduction**

Over the past 18 to 20 months you have been involved in the SCORE-IT project. Today I am here to get your feedback about your experience of participating in the SCORE implementation project. Your feedback is very important. We will use the results to implement evidence based stroke rehabilitation across Canada.

1. **Ground Rules**
   - No specific names of patients, coworkers, or hospital administrative personnel should be used.
   - Confidentiality: What we say in terms of specific people and their comments stays in this room. You should understand that your comments will be kept confidential by the project team as well.
   - Consent form: Each of you signed a consent form at the beginning of the research project agreeing that we can audio-record this session and that we can use the information and that your name is confidential and will never be used in any publications or presentations of this work. Do you still agree to this?

In addition, we would like to stress that you have been invited here to share your thoughts. There are no right or wrong answers. We want you to try and be as open and honest as you can be. We will start by discussing your personal experience with SCORE-IT and then we can discuss your overall impressions of implementing EBRs.

1. **Interview Questions:**

**Opening Question:** Let’s start by hearing from each of you about what happened during the SCORE-IT project.

Now we have some specific questions:

***Extent to which the proposed interventions were implemented:***

1. Tell me about your experiences implementing specific SCORE recommendations.

*Probes: What practices have changed?*

- - *Sit-to-stand*
  - *FES*
  - *Tasks for upper extremity*
  - *Intensive therapy (2 hours or more)*
  - *Aerobic exercises*
  - *Position of shoulder and shoulder pain*

*Which recommendations were easy to use in practice and why?*

1. Were there recommendations that your site did not implement? Can you give us examples and explain why?

*Probes:* *Which recommendations were difficult to use? Why?*

*Were there recommendations appropriate for a given patient that your team chose not to use? How did you make this decision?*

**Process of implementation at site:**

1. Did the implementation of the recommendations necessitate a significant change in practice? If so, can you provide examples?

*Probe: How did the recommendations change the way you and your team work (if at all)?*

1. Did any individual(s) assume a leadership or coordinating role of the implementation at your site? If so, can you describe what that person did to help the implementation?

**Barriers or facilitators to implementation:**

1. Describe any barriers in your practice environment to implementing the strategy at your site. *Probes:*
   - - *Presence of other studies*
     - *Time*
     - *Equipment*
     - *Communication*
     - *Space*
     - *Lack of support*

*What changes in your work environment would make it easier for you to use the recommendations that you found challenging to implement?*

1. In your opinion, how successful was the outcomes oriented strategy used by the SCORE team at your site in assisting you to implement the SCORE recommendations?

*Probes: Any feedback regarding the following?*

- 1. *DVD presentation*
  2. *Recommendations*

**Feedback for Improvement:**

1. What advice would you have for another centre thinking of implementing the SCORE recommendations or other clinical guidelines?
2. Any other final thoughts you would like to share with us?

We have a quick checklist that we would like you to complete and return.

We would like to thank you for your participation in today’s focus group and as well for all of your hard work throughout the past few months. We appreciate all the valuable time you have taken to participate in the SCORE-IT. We will summarize the thoughts and comments you have provided to explain the main findings of the study.

**D. Nurses Interview**

1. **Introduction**

Over the past 18 to 20 months you have been involved in the SCORE-IT project. Today I am here to get your feedback about your experience of participating in the SCORE implementation project. Your feedback is very important. We will use the results to implement evidence based stroke rehabilitation across Canada.

1. **Ground Rules**
   - No specific names of patients, coworkers, or hospital administrative personnel should be used.
   - Confidentiality: What we say in terms of specific people and their comments stays in this room. You should understand that your comments will be kept confidential by the project team as well.
   - Consent form: Each of you signed a consent form at the beginning of the research project agreeing that we can audio-record this session and that we can use the information and that your name is confidential and will never be used in any publications or presentations of this work. Do you still agree to this?

In addition, we would like to stress that you have been invited here to share your thoughts. There are no right or wrong answers. I want you to try and be as open and honest as you can be. We will start by discussing your personal experience with SCORE-IT and then we can discuss your overall impressions of implementing EBRs.

1. **Interview Questions:**

**Opening Question:** Let’s start by hearing from each of you about what happened during the SCORE-IT project.

Now we have some specific questions:

***Extent to which the proposed interventions were implemented:***

1. Tell me about your experiences implementing specific SCORE recommendations.

*Probes: What practices have changed?*

- - *Sit-to-stand*
  - *FES*
  - *Tasks for upper extremity*
  - *Aerobic exercises*
  - *Position of shoulder and shoulder pain*

*Which recommendations were easy to use in practice and why?*

1. Were there recommendations that your site did not implement? Can you give us examples and explain why?

*Probes:* *Which recommendations were difficult to use? Why?*

*Were there recommendations appropriate for a given patient that your team chose not to use? How did you make this decision?*

***Process of implementation at site:***

1. Did the implementation of the recommendations necessitate a significant change in practice? If so, can you provide examples?

*Probe: How did the recommendations change the way you and your team work (if at all)?*

1. Did any individual assume a leadership or coordinating role of the implementation at your site? If so, can you describe what that person did to help the implementation?

***Barriers or facilitators to implementation:***

1. Describe any barriers in your practice environment to implementing the strategy at your site. *Probes:*
   - - *Presence of other studies*
     - *Time*
     - *Equipment*
     - *Communication*
     - *Space*
     - *Lack of support*

*What changes in your work environment would make it easier for you to use the recommendations that you found challenging to implement?*

1. In your opinion, how successful was the outcomes oriented strategy used by the SCORE team at your site in assisting you to implement the SCORE recommendations?

*Probes: Any feedback regarding the following?*

- 1. *DVD presentation*
  2. *Recommendations*
  3. *Nursing Recommendation Poster*

***Feedback for improvement:***

1. What advice would you have for another centre thinking of implementing the SCORE recommendations or other clinical guidelines?
2. Any other final thoughts you would like to share with us?

We have a quick checklist that we would like you to complete and return.

We would like to thank you for your participation in today’s focus group and as well for all of your hard work throughout the past few months. We appreciate all the valuable time you have taken to participate in the SCORE-IT. We will summarize the thoughts and comments you have provided to explain the main findings of the study.
